# Editorial: Thalamic Interactions With the Basal Ganglia: Thalamostriatal System and Beyond

**DOI:** 10.3389/fnsys.2022.883094

**Published:** 2022-03-25

**Authors:** Jared B. Smith, Yoland Smith, Laurent Venance, Glenn D. R. Watson

**Affiliations:** ^1^Target Discovery, REGENXBIO Inc., Rockville, MD, United States; ^2^Yerkes National Primate Research Center, Emory University, Atlanta, GA, United States; ^3^Department of Neurology, Emory University, Atlanta, GA, United States; ^4^Udall Center of Excellence for Parkinson's Disease, Emory University, Atlanta, GA, United States; ^5^Center for Interdisciplinary Research in Biology (CIRB), College de France, CNRS, INSERM, Université PSL, Paris, France; ^6^Department of Psychology and Neuroscience, Duke University, Durham, NC, United States; ^7^LivaNova, Neuromodulation Unit, Houston, TX, United States

**Keywords:** thalamostriatal, basal ganglia, deep brain stimulation (DBS), thalamus, cortico-basal ganglia-thalamic

The basal ganglia have a long history of interest owing to their involvement across a wide array of neurological and psychiatric diseases (Redgrave et al., 2010). Much of the literature focuses on the role of the striatum, the main input nucleus to the basal ganglia, and its inputs from the cerebral cortex. Research on the role of thalamic inputs to the striatum has grown in recent years (Ding et al., 2010; Smith et al., 2014; Alloway et al., 2017; Assous et al., 2017; Unzai et al., 2017), as well as thalamic innervation of other basal ganglia nuclei (Deschenes et al., 1996; Mastro et al., 2014; Watson et al., 2021). In this special issue of Frontiers in Systems Neuroscience, we have collected a series of articles that illustrate the growing attention paid to the interactions between the thalamus and the basal ganglia. Two themes emerge from this collection. The first is a focus on more thoroughly elucidating the anatomy of the thalamus and the basal ganglia, including their connectivity; a topic that has also seen a renewed attention in the literature over the last decade with the advent of modern viral tracing methods in transgenic animals (Watabe-Uchida et al., 2012; Wall et al., 2013; Smith et al., 2016; Klug et al., 2018; Aoki et al., 2019; Foster et al., 2021; Lu et al., 2021; Watson et al., 2021). Along this theme, Kumar et al.; Kwon et al. employ magnetic resonance imaging (MRI) in high-strength magnetic fields to exquisitely dissect the anatomy of the thalamus and basal ganglia in the human brain. The second major theme of this special issue emerges from De Groote and de Kerchove d'Exaerde; Magnusson and Leventhal; Xiao and Roberts; Kato et al., which focus on the functional role of thalamic interactions with the basal ganglia in emotion, cognition, learning, attention, and other behavioral processes, as well as their role in disease.

## Anatomy of Thalamic Interactions With the Basal Ganglia

**Figure 1:**
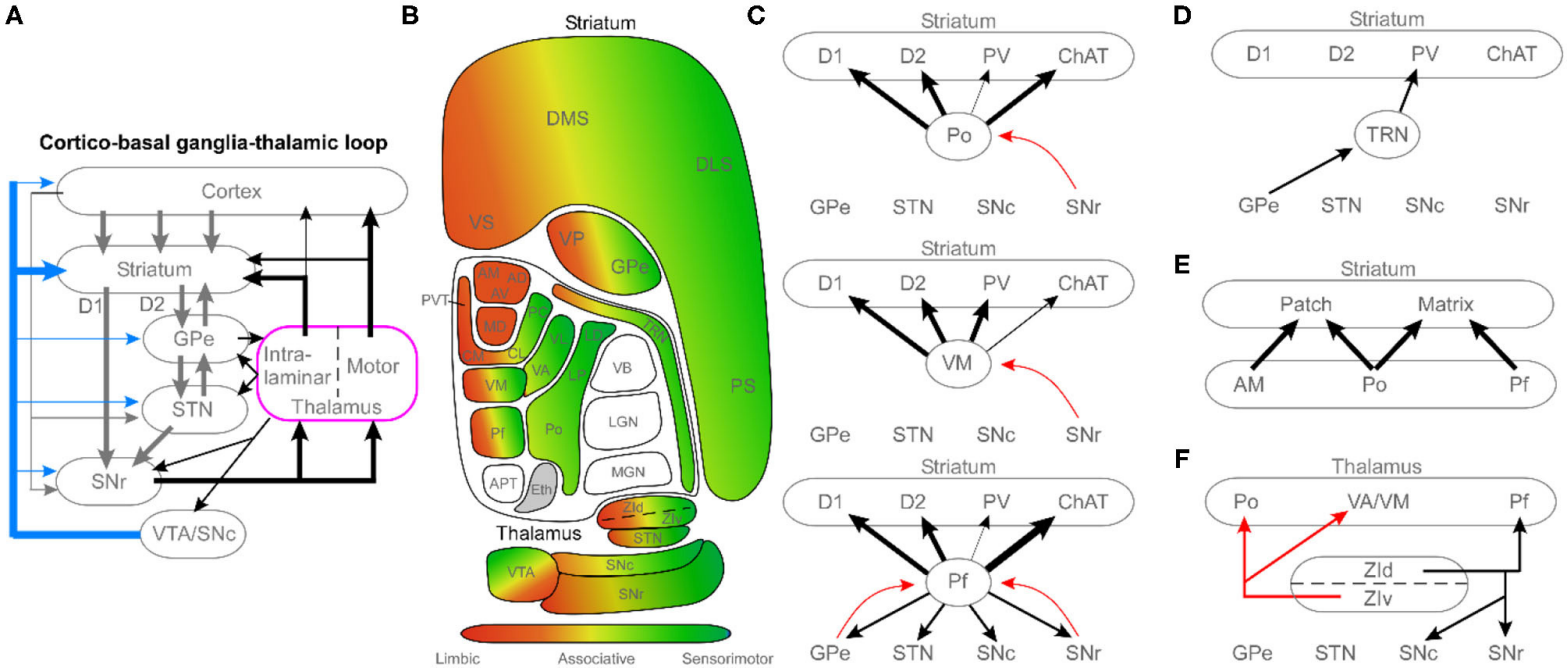
Circuit diagrams illustrating the complex diversity of thalamic connections with the basal ganglia. **(A)** Schematic of the cortico-basal ganglia-thalamic loop, highlighting the central role of the thalamus as a major recipient of basal ganglia output, and an important source of basal ganglia inputs. **(B)** Diagram of the topographic organization of the thalamus and basal ganglia, organized by limbic, associative, and sensorimotor regions. **(C)** Circuit diagrams illustrating differences among higher-order thalamic nuclei, motor thalamus, and caudal intralaminar thalamic nuclei. Diagrams feature cell-type specific innervation of the striatum (D1, direct pathway medium spiny neurons; D2, indirect pathway medium spiny neurons; PV, parvalbumin interneurons; ChAT, cholinergic interneurons) and unique patterns of connectivity with other basal ganglia nuclei. Black arrows, excitatory projections; red arrows, and inhibitory projections. **(D)** Connectivity of the thalamic reticular nucleus (TRN). **(E)** Relationship of three thalamic nuclei (limbic: AM; higher-order: Po; intralaminar: Pf) with respect to the patch (striosome) and matrix compartments of the striatum. **(F)** Connections of the zona incerta (ZI) that mediate interactions between the thalamus and basal ganglia. See the article text for references regarding the anatomical connectivity. Black arrows, excitatory projections; red arrows, inhibitory projections. APT, anterior pretectal nucleus; AD, anterodorsal nucleus; AM, anteromedial nucleus; AV, anteroventral; CM, centromedial nucleus; CL, centrolateral nucleus; DLS, dorsolateral striatum; DMS, dorsomedial striatum; Eth, ethmoid; GPe, globus pallidus external; LD, lateral dorsal nucleus; LGN, lateral geniculate nucleus; LP, lateral posterior; MGN, medial geniculate nucleus; Pf, parafascicular nucleus; PC, paracentral nucleus; PS, post-commissural striatum; PVT, periventricular thalamic nucleus; Po, posterior nucleus; SNc, substantia nigra pars compacta; SNr, substantia nigra pars reticulate; STN, subthalamic nucleus; TRN, thalamic reticular nucleus; VB, ventrobasal complex; VA, ventroanterior nucleus; VL, ventrolateral; VM, ventromedial nucleus; VP, ventral pallidum; VS, ventral striatum; VTA, ventral tegmental area; ZId, zona incerta dorsal; ZIv, zona incerta ventral.

Research on the cortico-basal ganglia-thalamic loop has largely viewed the thalamus as a relay that conveys basal ganglia output to the cerebral cortex to control movement. As shown in the circuit diagram in Figure 1A, recent anatomical tracing studies reveal a much more interactive relationship between the thalamus and the basal ganglia, wherein the thalamus has extensive input to the basal ganglia (primarily *via* projections to the striatum) in addition to receiving outputs from the substantia nigra pars reticulata (SNr), globus pallidus internal (GPi), and surprisingly the globus pallidus external (GPe). Using transgenic mice and viral-based tracing techniques, especially the g-deleted rabies technique, a more complex and nuanced set of connections have been described. From these tracing studies, several high-level organizational principles emerge.

First, Figure 1B illustrates the overall topography of limbic, associative, and sensorimotor regions across the thalamus and basal ganglia. Although many authors have focused on the segregated, parallel loop architecture of the cortico-basal ganglia-thalamic system (Mandelbaum et al., 2019; Foster et al., 2021), recent work has revealed a convergent, open-loop architecture across these modalities in addition to the closed loops (Aoki et al., 2019). The second major principle illustrated in Figure 1B is that not all thalamic nuclei interact directly with the basal ganglia. Specifically, primary sensory nuclei (lemniscal) project only to the cortex, with no input to the striatum (Alloway et al., 2017; Ponvert and Jaramillo, 2019). Thus, the bulk of thalamostriatal projections originate from the caudal intralaminar parafascicular nucleus (Pf), which projects preferentially to the striatum with modest cortical innervation as shown in Figure 1C. The remainder arise from thalamocortical collaterals from the rostral intralaminar, motor, and higher-order thalamic nuclei.

Finally, viral tracing studies have revealed a highly specific pattern of thalamic inputs to subtypes of striatal neurons (e.g., D1 and D2 medium spiny neurons, and parvalbumin and cholinergic interneurons). In addition, these studies suggest novel connections such as the thalamic reticular nucleus (TRN) input to striatal parvalbumin interneurons (Klug et al., 2018) shown in Figure 1D. They have also been useful for more carefully elucidating differences in the thalamic innervation of the striatal patch (striosome) and matrix compartments as shown in Figure 1E (see Raju et al., 2006; Unzai et al., 2017; Smith et al., 2016). Together, these rodent studies have identified a more complex thalamic interaction with the basal ganglia, which prompt the need for more non-human primate studies to learn if these projections are phylogenetically conserved in mammals that are more closely related to humans.

## Functional Role of Thalamic Interactions With the Basal Ganglia

Beyond anatomy, the modern armamentarium of systems neuroscience tools has provided new insights into the physiological and behavioral relevance of thalamostriatal interactions. As discussed by De Groote and de Kerchove d'Exaerde; Xiao and Roberts; Kato et al., the thalamostriatal synapse is uniquely positioned to facilitate learning and flexibility across limbic, cognitive, and sensorimotor modalities. The abundance of NMDA receptors and intralaminar inputs to cholinergic interneurons seem particularly poised to interact with corticostriatal and dopaminergic input; a critical substrate to support a host of motivated behaviors that includes sequence learning, such as vocalizations. In fact, *via* heterosynaptic interactions, thalamostriatal synaptic plasticity has recently been shown to shape the corticostriatal plasticity map, possibly enabling flexible behavior (Mendes et al., 2020).

Studies featured in this special issue also raise important questions about how to view the therapeutic role of thalamus-basal ganglia interactions. The review by Magnusson and Leventhal keenly discusses the problem of the traditional “rate model” view of the basal ganglia, as revealed by the paradox that both lesions and electrical excitement of nuclei within the basal ganglia are therapeutic in Parkinson's disease. Additionally, as shown in Figure 1F, a major role has emerged for the zona incerta (ZI) as a target for deep brain stimulation (DBS) based on its role as an interface between the thalamus and basal ganglia, including its profound inhibitory action on motor nuclei of the thalamus (Alloway et al., 2017; Ossowska, 2020). An early sign of things to come arises from one of our recent papers, showing that stimulation of functionally unexplored projections from Pf to STN, named the “super-direct” pathway, effectively rescues movement deficits in a Parkinsonian mouse model (Watson et al., 2021). By leveraging the nuanced anatomical connectivity between these structures, these emerging paradigms of the cortico-basal ganglia-thalamic system provide more accurate models that will undoubtedly be crucial for developing improved therapeutic strategies for basal ganglia-dependent neurological diseases.

## Author Contributions

JS constructed the figure. All authors drafted, revised, and approved final version of the editorial.

## Conflict of Interest

GW was employed by LIVANOVA. JS was employed by REGENXBIO Inc. The remaining authors declare that the research was conducted in the absence of any commercial or financial relationships that could be construed as a potential conflict of interest.

## Publisher's Note

All claims expressed in this article are solely those of the authors and do not necessarily represent those of their affiliated organizations, or those of the publisher, the editors and the reviewers. Any product that may be evaluated in this article, or claim that may be made by its manufacturer, is not guaranteed or endorsed by the publisher.
